# A Review of *in vitro* Platforms for Understanding Cardiomyocyte Mechanobiology

**DOI:** 10.3389/fbioe.2019.00133

**Published:** 2019-06-05

**Authors:** Ian L. Chin, Livia Hool, Yu Suk Choi

**Affiliations:** ^1^School of Human Sciences, The University of Western Australia, Perth, WA, Australia; ^2^Victor Chang Cardiac Research Institute, Sydney, NSW, Australia

**Keywords:** heart disease, biomaterials, hydrogels, cardiovascular disease, mechanosensation, elasticity, biophysical environment, extracellular matrix (ECM)

## Abstract

Mechanobiology—a cell's interaction with its physical environment—can influence a myriad of cellular processes including how cells migrate, differentiate and proliferate. In many diseases, remodeling of the extracellular matrix (ECM) is observed such as tissue stiffening in rigid scar formation after myocardial infarct. Utilizing knowledge of cell mechanobiology in relation to ECM remodeling during pathogenesis, elucidating the role of the ECM in the progression—and perhaps regression—of disease is a primary focus of the field. Although the importance of mechanical signaling in the cardiac cell is well-appreciated, our understanding of how these signals are sensed and transduced by cardiomyocytes is limited. To overcome this limitation, recently developed tools and resources have provided exciting opportunities to further our understandings by better recapitulating pathological spatiotemporal ECM stiffness changes in an *in vitro* setting. In this review, we provide an overview of a conventional model of mechanotransduction and present understandings of cardiomyocyte mechanobiology, followed by a review of emerging tools and resources that can be used to expand our knowledge of cardiomyocyte mechanobiology toward more clinically relevant applications.

## Introduction

Great progress has been made in understanding the various stimuli that regulate and maintain function in the beating heart. It is widely accepted that mechanical signaling is important for the coordination and regulation of cardiac function, and that the physical properties of the cardiac tissue change with aging and disease, however it remains unclear how the heart receives and responds to these mechanical signals.

Over the past decade, cell mechanobiology—the multidisciplinary study of interactions between cells and their physical environment—has illustrated that integrin-mediated sensation and transduction of mechanical signals plays an important role in controlling cellular behavior. New understandings from integrin-mediated mechanotransduction have contributed to advances in many fields, notably in stem cell technologies and understanding cancer progression. With respect to the importance of mechanical signaling in the heart, it is reasonable to suggest that interactions between cardiomyocytes and their physical environment would also be integral to the heart's function.

In this review, we provide a summary of the present understanding of cardiomyocyte mechanobiology, with a focus on integrin mediated mechanotransduction, and then provide an insight toward emerging tools and techniques that can be used to further expand understandings of cell mechanobiology in the heart.

## Foundations of Mechanobiology

### Mechanisms of Mechanosensation and Mechanotransduction

Cells receive many mechanical signals and cues from the extracellular matrix (ECM). These can include topographical variations in the substrate, the changes in stiffness over time and changes in stiffness over space. The ECM is formed from many proteins, glycosaminoglycans, and proteoglycans which all serve structural and non-structural roles in supporting and guiding cellular behavior. The components of the ECM and the roles thereof have been reviewed here (Bonnans et al., 2014). Notable protein constituents of the ECM are collagens, laminins, and fibronectins. These proteins act as ligands for cell adhesion as well as serving as being significant in the structure and organization of the ECM. The exact composition of the ECM varies from tissue to tissue, for example adipose tissue is rich in non-fibrillar collagen VI (Mariman and Wang, 2010) whereas cardiac tissue primarily consists of fibrillar collagen I. The composition of ECM can change over time, notably due to disease, which can result in marked remodeling of the ECM. Heart attack is an example of this, where, following a myocardial infarction, there is a dramatic increase in the proportion of collagen I at the site of the infarct as scar tissue is formed to replace dead cardiac tissue.

**Figure 1:**
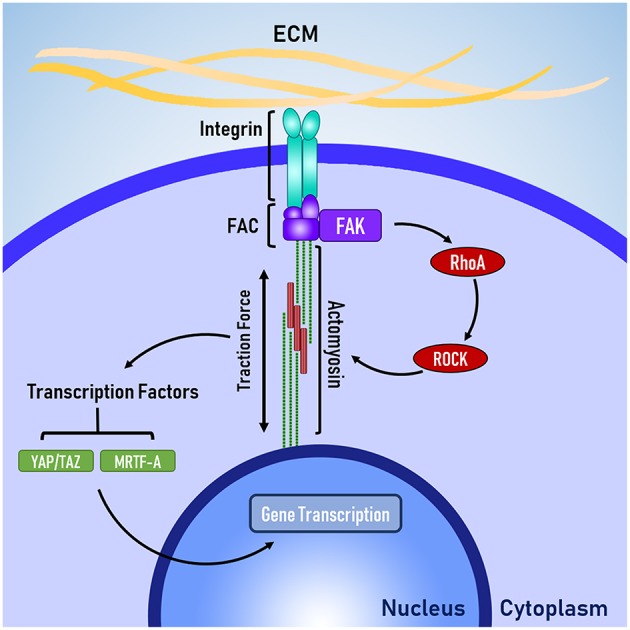
The basic mechanotransduction pathway. Mechanical forces transmitted through the ECM are received by the cell at integrins. Integrins are connected to the cytoskeleton via the focal adhesion complex (FAC). Adapter proteins within the FAC transduce mechanical signals in to biochemical signals, triggering RhoA-ROCK mediated contraction of non-muscle actomyosin. Traction forces are generated by actomyosin activity, which activate transcription factors that enact changes to gene transcription in the nucleus.

The ECM is key to carrying and providing mechanical cues to cells (Figure 1), which are in turn received by the cell through membrane bound integrins; heterodimers consisting of an alpha and beta subunit. Cellular interactions with the ECM can be mediated by the integrins that are expressed at the cell membrane. In mammals, there are 24 unique combinations of alpha and beta subunits, each binding to a specific set of ligands (Israeli-Rosenberg et al., 2014). Due to the specificity of integrins, the types and quantity of integrins on a cell membrane are believed to play a key role in determining the signals received by a cell and how the cell responds.

Integrins are connected to the cytoskeleton through a group of proteins known as the focal adhesion complex (FAC). FAC involves a number of adapter proteins, such as talin, α-actinin, and vinculin (Belkin and Koteliansky, 1987; Bois et al., 2006; Ye et al., 2014), which link integrins to filamentous actin of the cytoskeleton (Ye et al., 2014). These adapter proteins are believed to act like a “molecular clutch” because like a clutch in a car, these adapter proteins determine the level of engagement between the ECM and the cytoskeleton, which in turn determines how efficiently mechanical forces are transmitted between the ECM and the cytoskeleton (Swaminathan and Waterman, 2016). Talin and vinculin are believed to be mechanosensitive because talin has cryptic binding sites for vinculin, which are revealed through mechanical stretching (del Rio et al., 2009) and vinculin appears to have force-mediated recruitment and activation (Balaban et al., 2001) via mitogen activated protein kinase (MAPK) binding (Holle et al., 2013).

The FAC can be a site of signal transduction, where mechanical signals are transformed into biochemical signals. One of the key signal-transduction pathways is the RhoA-ROCK pathway, where focal adhesion kinase (FAK) phosphosrylates RhoA, which leads to the activation of Rho associated protein kinase 1 (ROCK). FAK has also been implicated heavily in the development of cancer and researchers are currently investigating whether FAK could be a therapeutic target for treating various forms of cancer (Lee et al., 2015; Hirt et al., 2018). Laboratory studies have identified several promising inhibitors, which are progressing through the early stages of clinical testing (Lee et al., 2015; Lv et al., 2018).

The RhoA-ROCK pathway is responsible for cellular force generation through the phosphorylation of myosin light chain, which leads to contraction of non-muscle actin-myosin filaments generation traction forces. Traction forces exerted by the cell are resisted by the cellular microenvironment, which leads to changes in cell shape and controls the level of force applied to the membrane-bound integrins. Because the generation of traction forces are mediated by the physical properties of the cellular microenvironment (e.g., topography, stiffness, and ligand availability), it is believed that the generation of tractions forces is a mechanisms for cells to actively probe and respond the mechanical properties of their microenvironment (Lo et al., 2000; Ingber, 2003; Eyckmans et al., 2011). Beyond the generation of traction forces, the RhoA-ROCK pathway has been implicated in several other mechanosensitive processes, such as the translocation of the co-transcription factors YAP/TAZ (Dupont et al., 2011) and MRTF-A (Yu et al., 2016), which shuttle between the cytoplasm and the nucleus to signal changes in gene transcription.

### Protein Markers of Mechanosensation

**Figure 2:**
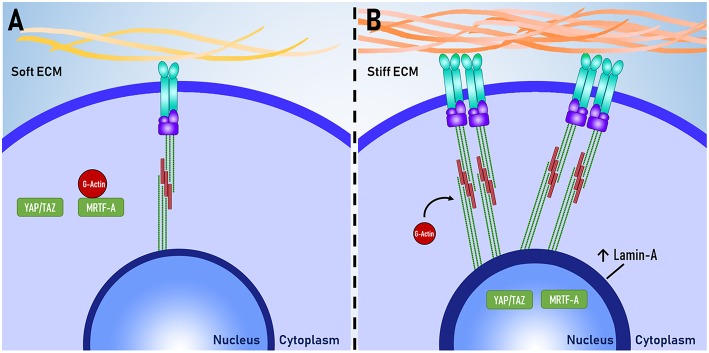
Proteins as mechanomarkers. Expression of Lamin A, YAP and MRTF-A are dependent on mechanical signaling. When exposed to **(A)** soft ECM, YAP/TAZ and MRTF-A are inactive. When exposed to a **(B)** stiff ECM, there is increased assembly of non-muscle actomyosin stress fibers and focal adhesion complexes. MRTF-A dissociates from globular actin (G-actin) and both YAP/TAZ and MRTF-A translocate to the nucleus. The expression of Lamin-A in the nuclear lamina also increases.

A number of proteins that are sensitive to changes in the mechanical environment have been identified and it has been suggested that these proteins could be used to better understand the relationship between cellular functions and mechanosensation. Using immunocytochemistry, several studies have quantified the expression of these proteins and used protein expression as a measure of mechanosensation. Key proteins are described below, and their mechanosensitive expression is visually illustrated in Figure 2.

#### Lamin-A

Lamin-A is one of the intermediate filaments that constitute the nuclear lamina. Lamin-A is believed to play a key role in determining nuclear stiffness and to have a regulatory role in gene expression through interactions between DNA and the nuclear lamina (Swift et al., 2013). Lamin-A is also essential for post-natal growth as knockout of lamin-A in mouse models results in severe growth retardation and premature death (Sullivan et al., 1999; Jahn et al., 2012). Lamin-A expression has been used an indicator of mechanosensation as Lamin-A expression scales with tissue stiffness *in vivo* and substrate stiffness *in vitro*, whereby increased substrate stiffness results in increased lamin-A expression (Swift and Discher, 2014).

#### YAP/TAZ

Yes-associated protein (YAP) and transcriptional coactivator with PDZ-binding motif (TAZ) are co-transcription factors that have been implicated as major regulators of mechanotransduction. YAP/TAZ are believed to relay mechanical signals from the cytoplasm to the nucleus, where they enact changes in gene expression. Several experiments have shown that increased traction force increases nuclear YAP localization and that inhibition of mechanotransduction abolishes this response (Dupont et al., 2011; Hadden et al., 2017). YAP/TAZ localization is useful as a tool for measuring the level of mechanotransduction within a cell.

#### MRTF-A

Myocardin related transcription factor A (MRTF-A), also known as MKL1, is a transcription factor that shuttles between the cytoplasm and the nucleus. When inactive, MRTF-A is bound to globular actin (G-actin) and is localized within the cytoplasm. MRTF-A becomes active when G-actin dissociates from MRFT-A to be polymerised to form actomyosin. Upon dissociation, MRTF-A is sequestered in to the nucleus where it can enact changes to gene expression (Yu et al., 2016). Similar to YAP/TAZ, the degree of MRTF-A nuclear or cytoplasmic localization can be used as an indicator of mechanosensation (Hadden et al., 2017).

### Mechanical Cues Control Major Cellular Processes

Traditionally, mechanobiology has been studied using 2-dimensional (2D), spatially and temporally static hydrogel platforms. Hydrogels have been made from naturally derived materials, such as collagen or hyaluronic acid (HA), and synthetic materials, such as polyacrylamide (PA) and polyethylene glycol (PEG). The advantages of each material has been debated in past and has been reviewed in more detail here (Ruedinger et al., 2014; Thiele et al., 2014), however all have been used to reveal foundational insights toward cell mechanobiology.

In many cell types, morphology, apopotosis, and proliferation are controlled through mechanosensation. Conventionally on a 2D substrate, cells are small and circular on a soft matrix and large and spread on a stiff matrix (Wang et al., 2000; Engler et al., 2006; Hadden et al., 2017) and in fibroblasts cultured on collagen hydrogels (Hadjipanayi et al., 2009) or collagen coated PA (Wang et al., 2000) increased rates of proliferation and decreased rates of apoptosis were observed on stiffer substrates.

Mechanical signaling is involved differentiation and migration. An early demonstration of this used PA hydrogels of different substrate stiffness to guide the differentiation of mesenchymal stem cells (MSC) cultured in identical serum conditions (Engler et al., 2006). Using early markers of lineage specification, it was found that cells preferentially differentiated toward tissues that were similar in stiffness to the substrates they were cultured on. For example, a soft matrix (<1 kPa) promoted neurogenic differentiation whereas a stiff matrix (>25 kPa) promoted osteogenic differentiation. It has been shown that similar outcomes can be achieved by controlling cell shape (McBeath et al., 2004; Kilian et al., 2010; Major and Choi, 2018), whereby increasing cell size promoted osteogenic differentiation and smaller cell size promoted adipogenic differentiation. Stiffness-mediated and shape-mediated changes to cell behavior are believed to both be a consequence of traction force generation, as traction forces can be controlled through both substrate stiffness and cell shape (Califano and Reinhart-King, 2010; Rape et al., 2011).

Further studies demonstrated that many cells, including adipose-derived stem cells (ASC) (Hadden et al., 2017), MSCs (Vincent et al., 2013), and fibroblasts (Hartman et al., 2017) preferentially migrate toward stiffer substrates through the process of durotaxis. Durotaxis has been demonstrated through time lapse microscopy of cells cultured on hydrogels patterned with a stiffness gradient. Durotaxis is mediated by ECM ligands that the cells are exposed to Hartman et al. (2017) and has been shown in ASC to only occur above a threshold stiffness gradient (8.2 kPa/mm) (Hadden et al., 2017).

## Current understanding of cardiac mechanobiology

### Cardiomyocytes Are Mechanosensitive

In the context of cardiac physiology, it is accepted that cardiomyocytes are mechanosensitive, however the mechanisms and implication of cardiomyocyte mechanosensation are not well-understood.

There is evidence that appropriately matching substrate stiffness can promote the maturation of induced pluripotent stem cell (iPSC) derived cardiomyocytes (Herron et al., 2016) and of embryonic cardiomyocytes (Young and Engler, 2011). Cardiomyocytes derived from iPSC and cultured on polydimethylsiloxane (PDMS) membranes had more mature electrophysiology compared to cells cultured on glass, shown by action potential configuration and conduction, and connexin 43 expression. Embryonic chicken cardiomyocytes demonstrated increased levels of the late cardiac marker troponin-T and a time-dependent decrease in the early cardiac marker NKX2.5 when cultured on a modified hyaluronic acid (HA) that stiffened over time as opposed to cells cultured temporally static hydrogels (Young and Engler, 2011). In conjunction with the observations from derived cardiomyocytes, it appears that substrate stiffness plays an important role in the maturation of cardiac muscle and that these changes are time dependent.

Substrate stiffness is also known to alter the phenotype of cardiomyocytes. In embryonic quail and neonatal rat cardiomyocytes grown on 2D polyacrylamide (PA) hydrogels, cell shape and the level of sarcomere organization were found to be stiffness dependent (Engler et al., 2008; Jacot et al., 2008). In both cases, it was found that approximately 10 kPa was the optimal stiffness for the growth of cardiomyocytes, where the shape was more myocyte-like, the sarcomeres showed clear striations and functionally cells were better capable of maintaining constant spontaneous beating.

Substrate stiffness also appears to play an important role in force generation during contraction (Hersch et al., 2013; Ribeiro et al., 2015). Hersch et al. used traction force microscopy to measure the force of contraction of single rat cardiomyocytes cultured on PDMS substrates of varying stiffness. As substrate stiffness increased, the length of contraction remained constant and the force of contraction increased accordingly (Hersch et al., 2013). In this study, the authors did not observe any structural differences between cardiomyocytes cultured on different substrate stiffnesses, potentially because late embryonic cardiomyocytes were used which may have been more terminally differentiated than cardiomyocytes used in other studies. Based on these observations, the authors suggested that cardiomyocytes may be able to quickly adapt the force of contraction in response to mechanical resistance from the cellular microenvironment without significant reorganization of the cytoskeleton.

There appear to be two main pathways for mechanotransduction in cardiomyocytes: through integrins and through cadherins. Chopra et al. demonstrated this by culturing neonatal rat cardiomyocytes on PA gels coated in either type I collagen or in N-cadherin, neonatal rat cardiomyocytes demonstrated many similar trends in cell shape, cytoskeletal organization and the generation of traction forces (Chopra et al., 2011). N-cadherin is a major constituent of the intercalated discs, a strong mechanical linkage between cardiomyocytes, and has been shown to be essential for the development of the heart (Kostetskii et al., 2005). The intercalated disc is a characteristic feature of mature cardiomyocytes not typically found in the mesenchymal cells used to model mechanotransduction. This study suggested that cardiomyocytes are sensitive to N-cadherin mediated mechanical signaling and possibly also cell-cell mechanical signaling through the intercalated discs. Other studies have suggested that the loss of intercalated disks plays a role in age-related loss of cardiac function and that reinforcement of the intercalated disks may help preserve cardiac function (Kaushik et al., 2015; Sessions et al., 2018).

### The Cardiac Cellular Microenvironment

**Figure 3:**
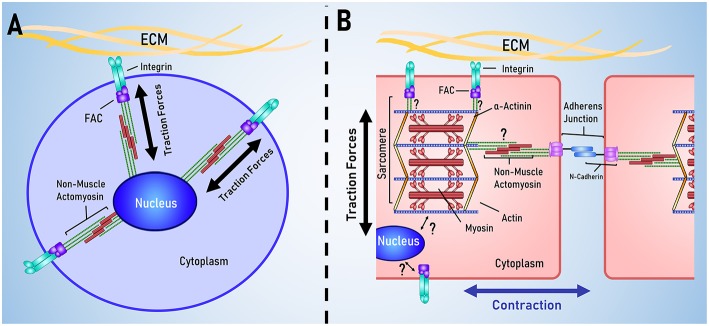
Key differences between stem cell and cardiac mechanotransduction. **(A)** In stem cell models of mechanotransduction, mechanical signals are received by membrane bound integrins, transduced in to biochemical signals that result in traction force generation by non-muscle actomyosin. **(B)** Cardiomyocytes are characterized by having aligned muscle actomyosin and intercalated disks; intercellular links for electrical and mechanical coordination of contraction. It is currently unclear how the sarcomere and nucleus are mechanically linked to membrane bound integrins and the cadherins that link cells together.

The heart is unique as an organ in the way that it spontaneously contracts in a coordinated fashion. Accordingly, cardiomyocytes are unique in their cellular structure and organization to support the function of the heart. Mechanotransduction is often modeled using stem cells, however there are several key structural differences between cardiomyocytes and stem cells, outlined in Figure 3.

Like most ECM, the cardiac ECM is constituted of many components, and has been reviewed here (Nielsen et al., 2017), however the key elements are summarized here. Structurally, the ECM can be divided in to the interstitial matrix, the ECM that exists between cells, and the basement membrane, the ECM that immediately surrounds the cells. In the heart, the interstitial matrix is comprised mostly of fibrillar collagen I, and primarily provides structural support for the cell. The basement membrane contains a mixture of proteins including laminin, collagen, and many proteoglycans. Throughout the ECM are non-structural matricellular proteins, such as osteopontin and matrix metalloproteins, which help in organizing the structure of the ECM and can provide important chemical signals to cells. With regards to the physical properties of healthy cardiac tissue, the stiffness is typically around 10 kPa (Berry et al., 2006; Janmey and Miller, 2011). Following myocardial infarction, tissue stiffness changes dramatically at the site of the infarct, initially softening as dead cells are removed and then increasing to approximately 40 kPa during scar formation (Berry et al., 2006). *In vitro* matching substrate stiffness to physiological values (~10 kPa) has been found to promote maturation and improve contractility in neonatal cardiomyocytes whereas pathological stiffnesses (>35 kPa) result in hypertrophy and reduced contractility (Engler et al., 2004; Jacot et al., 2008; McCain et al., 2014).

Cardiomyocytes adhere to ECM primarily through α1β1, α5β1, and α7β1 integrins, which bind to collagen, fibronectin, and laminin, respectively (Israeli-Rosenberg et al., 2014). Integrin expression varies as a consequence of development and disease. For example, the expression of the α5 subunit is reduced and the expression of the α7 subunit increases during post-natal development (Brancaccio et al., 1998) and ischemia can promote the expression of both the α5 and α7 subunits (Nawata, 1999).

The cellular structure and organization of cardiomyocytes have been reviewed in depth by Lyon et al. (2015) and so will be only briefly covered by this review. There are two key function-specific structures in cardiomyocytes: the sarcomere and the intercalated disk. The sarcomere is the basic contractile unit within the cardiomyocyte and the degree of sarcomeric organization is often used as an indicator of cardiomyocyte differentiations and maturation. Sarcomeres are often visualized using α-actinin (Ribeiro et al., 2015; Pandey et al., 2018), troponin-I (Annabi et al., 2013; Li et al., 2016) and troponin-T (Yahalom-Ronen et al., 2015; Li et al., 2017) and other sarcomeric proteins, such as myosin heavy chain and myosin light chain, are used as indicators of cardiac differentiation and maturation (Choi et al., 2010; Higuchi et al., 2013; Yahalom-Ronen et al., 2015; Li et al., 2017). As traction forces play a significant role in mechanosensation, it is unclear how the additional contractile apparatus affects the rest of mechanotransduction pathway.

The intercalated disc is a characteristic feature of mature cardiomyocytes not typically found in the mesenchymal cells used to model mechanotransduction. Intercalated discs form the major link between adjacent cardiomyocytes and are essential for coordinating contraction and transmitting electrical and mechanical signals between cells. N-cadherin is a major component of intercalated discs, being involved in the formation of fascia adherens junctions and area composita, two junctions that mechanically link the intercalated disc to the cytoskeleton (Mezzano and Sheikh, 2012). In mouse models, knockout of N-cadherin leads to a loss of intercalated discs, morphological changes to the heart that resemble a dilated cardiomyopathy and leads to sudden death (Kostetskii et al., 2005). There is evidence that cadherins can mediate mechanotransduction independent of integrins. Chopra et al. demonstrated this by culturing neonatal rat cardiomyocytes on PA gels coated in either type I collagen or in N-cadherin, neonatal rat cardiomyocytes demonstrated many similar trends in cell shape, cytoskeletal organization and the generation of traction forces (Chopra et al., 2011). This study suggested that cardiomyocytes are sensitive to N-cadherin mediated mechanical signaling and possibly also cell-cell mechanical signaling through the intercalated discs.

## Emerging Materials and Methods for Understanding Cardiac Mechanobiology

Whilst simple platforms have provided us with a basic understanding of cardiac mechanobiology, the *in vivo* cardiac environment combines a number of complex mechanical signals. Cardiac tissue is striated, temporally dynamic, 3-dimensional (3D), and viscoelastic. To emulate this, materials would need to incorporate spatial patterning, temporal patterning, support 3D culture, have tuneable viscoelastic elements and furthermore would also need to combine all these elements to reveal how each signal interacts with one another. Similar challenges are faced across the field of mechanobiology and to this end, new materials and methods have been developed that can allow for more comprehensive mimicry of the cellular microenvironment. To bridge the gap between *in vitro* platforms and the *in vivo* environment, we should take inspiration from these materials and methods used to study mechanobiology in other cells, such as stem cells, fibroblasts or cancer cells, and adapt them to expand upon our understanding of cardiomyocyte mechanobiology.

### Spatial Patterning

Recent developments in spatially patterned platforms have presented some interesting solutions to some of the challenges in studying cardiomyocytes. A number of techniques have been developed to create platforms with a stiffness gradient, as a high-throughput system for analyzing mechanosensitive properties of cells (Hartman et al., 2016; Hadden et al., 2017). These platforms were used to generate higher resolution data than past studies by examining protein expression, cell morphology, and cell migration on a continuous gradient rather than at discrete stiffnesses. These platforms were developed using PA and took utilized chemical gradients during fabrication to generate a mechanical gradient, however there are other approaches to developing patterned hydrogels. A promising approach is to use photolithography, where controlled light exposure is used to create mechanical patterns in hydrogels during fabrication. This can be achieved using hydrogels with a photoinitiated fabrication process, such as gelatin methacyloyl (GelMA), where a photomask can be used to vary light exposure across the hydrogel during fabrication, allowing for a stiffness pattern to be incorporated in to the hydrogel.

Spatial patterning of hydrogels can also involve controlling the area available for cells to grow. In the past, this has involved microcontact stamping, to control the spatial distribution of ligands across the substrate (Engler et al., 2004) or microcontact printing, to control the physical area available for cell adhesion (McBeath et al., 2004; Kilian et al., 2010; Major and Choi, 2018). Typically, microcontact printing involves fabricating PDMS stamps that are used to define an area where an ECM protein, such as fibronectin, could be applied allowing cells to adhere to the area marked by the PDMS stamp, but not elsewhere. In the past, this technique has been primarily used to guide differentiation, in mixed differentiation media, by controlling cell shape. McBeath et al. found that restricted cell spreading promoted adipogenesis and larger spreading areas promoted osteogenesis (McBeath et al., 2004). Kilian et al. confirmed this result and found that the key to guiding differentiation was cell contractility, measured through myosin IIa expression, rather than cell size itself (Kilian et al., 2010). Major and Choi further investigated cell spreading and size work in conjunction with substrate stiffness to determine differentiation, however this study found that under more complex conditions, signals can compete and mask one another. For example, under adipogenic stiffness conditions, increasing cell spreading area would promote the expression of the osteogenic differentiation marker RUNX2 however when cultured on osteogenic stiffness conditions, cell size-induced differences in RUNX2 expression were attenuated. This illustrates the potential complications using complex platforms as well as the importance of developing and studying combinatorial platforms.

Patterning hydrogels like this allows the distribution of cells to be controlled, making it possible to examine the differences between cell-ECM and cell-cell signaling in cardiomyocyte mechanobiology. In light of the known importance of intercalated discs in cardiac function (Sheikh et al., 2009; Lyon et al., 2015) and the differences between integrin and N-cadherin mediated mechanosensation (Chopra et al., 2011), it would be valuable to understand how different forms of mechanical signaling effects cardiomyocytes and the implications this might have if the relationship between the cells and their microenvironment were to change.

### Temporal Patterning

Given that many diseases change the stiffness of the ECM over time, it is possible that the ECM could be a target for treatments. This concept has been investigated in the context of cancer, however the importance of time-dependent stiffness changes is not well-understood. Temporal elements in hydrogel platforms are invaluable in understanding the importance of time dependent changes as they reveal the interplay between mechanical signals across time, something which is not captured by temporally static platforms.

A major limitation of many dynamically stiffened hydrogels is that the stiffness changes are irreversible and so cannot investigate the reversibility of stiffness-induced cellular changes. Rosales et al. (2017) overcame this challenge by developing a material that combined two independent chemistries; one chemistry that is photodegradable and one chemistry for photocrosslinking, so that the material could be both softened and stiffened on demand. Using this material, the investigators were able to soften the material from 14.8 to 3.5 kPa and then stiffen the material to 27.7 kPa, illustrating the range of stiffnesses achievable by the material. Using this platform, the investigators probed the capacity of human MSCs to respond to dynamic environmental stiffness changes, chiefly examining cell size, shape and nuclear YAP localization. It was found that, as demonstrated by temporally static platforms, that reducing substrate stiffness reduced cell size, increased the roundness of the cells and decreased the level of nuclear YAP localization and the opposite for increasing substrate stiffness. Interestingly, whilst increasing substrate stiffness did partially restore cell size, shape and YAP localization to pre-softening levels, complete recovery was not observed despite the post-stiffening stiffness being double that of the initial stiffness. These findings suggest that there are time-dependent characteristics of mechanosensation, not captured by static hydrogel platforms.

These observations are supported by Abdeen et al. (2016), who performed a similar experiment using a hydrogel material functionalised with magnetic particles. Using their magnetoactive hydrogel, substrate stiffness can be reversibly increased from 0.1 to 90 kPa by applying a magnetic field to the hydrogel. Investigators measured cell size and Runx2 expression as cells were exposed to various regimes of substrate softening and stiffening and similar to Rosales et al. it was observed that the relationship between cell size and Runx2 expression and substrate stiffness was dependent on the regime the cells were exposed to.

Findings from these temporally dynamic platforms has illustrated that the dimension of time is an important consideration for the study of mechanobiology and potentially treatments targeting the ECM. With regards to heart disease, understanding time-dependent changes to cardiac mechanobiology would inform us of appropriate time frames for treatments or the suitability of certain treatments, given the degree of ECM remodeling observed in disease, such as myocardial infarction coupled with limitations in our capacity to alter the physical characteristics of the *in vivo* environment.

### Dimensionality

Introducing dimensionality has been a large focus of recent works as it is generally appreciated that many cells do not grow in a 2D monolayer but in a 3D environment, however until recently there were few platforms that were suitable or able to sustain a 3D culture. There are now several materials that are available for encapsulating cells, such as GelMA (Loessner et al., 2016) and modified HA (Caliari et al., 2016), which have shown that the trends observed in 2D are not necessarily representative of the trends observed in 3D. Notably, cell spreading and YAP/TAZ nuclear localization were reduced as stiffness increased in 3D, which is the inverse of what is traditionally observed in 2D (Caliari et al., 2016). Possible explanations to this could be related to the pore size of the material, which is coupled to the stiffness of the material and could be restricting the volume expansion of cells, which has been suggested as a key regulator of cellular functions (Lee et al., 2019). This idea is supported by Caliari et al. who found that increasing the susceptibility of the hydrogel to enzymatic degradation enhanced cell spreading and the nuclear translocation of YAP (Caliari et al., 2016), suggesting that cell volume itself may play a key role in regulating cellular function.

### Viscoelasticity

Many hydrogel materials used for past *in vitro* studies, such as PA, are purely elastic, however for many tissues, including myocardium, the ECM is actually viscoelastic (Urban et al., 2013; Wang et al., 2016). As a viscoelastic material, the ECM has time-dependent characteristics in the way that it responds to stress (force applied per unit of area) and strain (the degree to which an object is deformed). These properties are generally described by strain-rate dependence, stress-relaxation and creep, which are best explained by engineering textbooks such as “Mechanical Response of Polymers: An Introduction” (Wineman and Rajagopal, 2000). Presently, the viscoelasticity in many hydrogel materials has not been well-characterized and there are few materials where viscoelastic properties can be tuned, however based on the materials that are available, it has been shown that viscoelasticity can have a significant effect on cell behavior and growth.

In one example, a modified alginate-based hydrogel material was used to investigate the role of stress-relaxation in stem cell differentiation. The material had mobile crosslinks that become restructured as stresses and strains were applied, and could be modified to tune the stress-relaxation time of the material. In samples with the same initial elastic modulus, it was found that the stress relaxation time mediated cell spreading in 3D and could guide stem cell differentiation synergistically with other differentiation cues (Chaudhuri et al., 2016; Lee et al., 2017). In another study, PA was used to study viscoelasticity, through varying the proportions of acrylamide and bis-acrylamide to generate gels with the same elasticity but different creep responses (Cameron et al., 2011) and it was found that creep influenced the morphology, proliferation and differentiation potential of human MSCs.

Viscoelasticity is a property of biomaterials that in past has had limited exploration due to limitations of the materials available, however emerging materials have highlighted that cells can detect and respond the viscoelasticity of their environment. This makes viscoelasticity an important consideration in trying to understand mechanosensation and mechanotransduction, notably in 3D culture environments, and is another facet of the cellular microenvironment that can be emulated.

## From Benchtop to Bedside

Many innovative and creative strategies have been devised to overcome the challenges involved in mimicking the *in vivo* environment *in vitro*. This has involved adapting simple materials to be used in complex applications and opening new opportunities through the development of new materials, allowing us greater control over the way we mimic the spatial and temporal elements of the cellular microenvironment in 2D and 3D. With these developments in mind, there are now many resources available for study the role of mechanosensation in the transition from healthy to pathological states and the possibility of manipulating the physical environment, or cells perception thereof, as a form of treatment. There is also potential in devising combinatorial platforms that combine biochemical, electrical and mechanical signaling in order enhance the efficacy of *in vitro* studies.

Major challenges still exist, notably difficulties in studying viscoelasticity and understanding the discrepancies between mechanosensation in 2D and 3D. Despite this, the frontier of our mechanobiology understandings have expanded through the development of tools and resources, and in bridging the gap between *in vitro* studies and *in vivo* observations.

## Author Contributions

All authors listed have made a substantial, direct and intellectual contribution to the work, and approved it for publication.

### Conflict of Interest Statement

The authors declare that the research was conducted in the absence of any commercial or financial relationships that could be construed as a potential conflict of interest.

## References

[B1] Abdeen A. A. Lee J. Bharadwaj N. A. Ewoldt R. H. Kilian K. A. (2016). Temporal modulation of stem cell activity using magnetoactive hydrogels. Adv. Healthc. Mater. 5, 2536–2544. 10.1002/adhm.201600349 27276521 PMC5061612

[B2] Annabi N. Tsang K. Mithieux S. M. Nikkhah M. Ameri A. Khademhosseini A.. (2013). Highly elastic micropatterned hydrogel for engineering functional cardiac tissue. Adv. Funct. Mater. 23, 4950–4959. 10.1002/adfm.201300570 24319406 PMC3850066

[B3] Balaban N. Q. Schwarz U. S. Ishizaki T. Narumiya S. (2001). Focal contacts as mechanosensors externally applied local mechanical force induces growth of focal. J. Cell Biol. 153, 1175–1185. 10.1083/jcb.153.6.1175 11402062 PMC2192034

[B4] Belkin A. M. Koteliansky V. E. (1987). Interaction of iodinated vinculin, metavinculin and α-actinin with cytoskeletal proteins. FEBS Lett. 220, 291–294. 10.1016/0014-5793(87)80832-3 3111888

[B5] Berry M. F. Engler A. J. Woo Y. J. Pirolli T. J. Bish L. T. Jayasankar V.. (2006). Mesenchymal stem cell injection after myocardial infarction improves myocardial compliance. Am. J. Physiol. Heart Circ. Physiol. 290, H2196–H2203. 10.1152/ajpheart.01017.2005 16473959

[B6] Bois P. R. O'Hara B. P. Nietlispach D. Kirkpatrick J. Izard T. (2006). The vinculin binding sites of talin and α-actinin are sufficient to activate vinculin. J. Biol. Chem. 281, 7228–7236. 10.1074/jbc.M510397200 16407299

[B7] Bonnans C. Chou J. Werb Z. (2014). Remodelling the extracellular matrix in development and disease. Nat. Rev. Mol. Cell Biol. 15, 786–801. 10.1038/nrm3904 25415508 PMC4316204

[B8] Brancaccio M. Cabodi S. Belkin A. M. Collo G. Tomatis D. Altruda F.. (1998). Differential onset of expression of ?7 and ?1D integrins during mouse heart and skeletal muscle development. Cell Adhes. Commun. 5, 193–205. 10.3109/15419069809040291 9686317

[B9] Caliari S. R. Vega S. L. Kwon M. Soulas E. M. Burdick J. A. (2016). Dimensionality and spreading influence MSC YAP/TAZ signaling in hydrogel environments. Biomaterials 103, 314–323. 10.1016/j.biomaterials.2016.06.061 27429252 PMC4963302

[B10] Califano J. P. Reinhart-King C. A. (2010). Substrate stiffness and cell area predict cellular traction stresses in single cells and cells in contact. Cell. Mol. Bioeng. 3, 68–75. 10.1007/s12195-010-0102-6 21116436 PMC2992361

[B11] Cameron A. R. Frith J. E. Cooper-White J. J. (2011). The influence of substrate creep on mesenchymal stem cell behaviour and phenotype. Biomaterials 32, 5979–5993. 10.1016/j.biomaterials.2011.04.003 21621838

[B12] Chaudhuri O. Gu L. Klumpers D. Darnell M. Bencherif S. A. Weaver J. C.. (2016). Hydrogels with tunable stress relaxation regulate stem cell fate and activity. Nat. Mater. 15, 326–334. 10.1038/nmat4489 26618884 PMC4767627

[B13] Choi Y. S. Matsuda K. Dusting G. J. Morrison W. A. Dilley R. J. (2010). Engineering cardiac tissue *in vivo* from human adipose-derived stem cells. Biomaterials 31, 2236–2242. 10.1016/j.biomaterials.2009.11.097 20031204

[B14] Chopra A. Tabdanov E. Patel H. Janmey P. A. Kresh J. Y. (2011). Cardiac myocyte remodeling mediated by N-Cadherin-dependent mechanosensing. AJP Heart Circ. Physiol. 300, H1252–H1266. 10.1152/ajpheart.00515.2010 21257918 PMC3075038

[B15] del Rio A. Perez-Jimenez R. Liu R. Roca-Cusachs P. Fernandez J. M. Sheetz M. P. (2009). Stretching single talin rod molecules activates vinculin binding. Science 323, 638–641. 10.1126/science.1162912 19179532 PMC9339221

[B16] Dupont S. Morsut L. Aragona M. Enzo E. Giulitti S. Cordenonsi M.. (2011). Role of YAP/TAZ in mechanotransduction. Nature 474, 179–184. 10.1038/nature10137 21654799

[B17] Engler A. J. Carag-Krieger C. Johnson C. P. Raab M. Tang H. Y. Speicher D. W.. (2008). Embryonic cardiomyocytes beat best on a matrix with heart-like elasticity: scar-like rigidity inhibits beating. J. Cell Sci. 121, 3794–3802. 10.1242/jcs.029678 18957515 PMC2740334

[B18] Engler A. J. Griffin M. A. Sen S. Bönnemann C. G. Sweeney H. L. Discher D. E. (2004). Myotubes differentiate optimally on substrates with tissue-like stiffness: pathological implications for soft or stiff microenvironments. J. Cell Biol. 166, 877–887. 10.1083/jcb.200405004 15364962 PMC2172122

[B19] Engler A. J. Sen S. Sweeney H. L. Discher D. E. (2006). Matrix elasticity directs stem cell lineage specification. Cell 126, 677–689. 10.1016/j.cell.2006.06.044 16923388

[B20] Eyckmans J. Boudou T. Yu X. Chen C. S. (2011). A Hitchhiker's guide to mechanobiology. Dev. Cell 21, 35–47. 10.1016/j.devcel.2011.06.015 21763607 PMC3155761

[B21] Hadden W. J. Young J. L. Holle A. W. McFetridge M. L. Kim D. Y. Wijesinghe P.. (2017). Stem cell migration and mechanotransduction on linear stiffness gradient hydrogels. Proc. Natl. Acad. Sci. USA. 114, 5647–5652. 10.1073/pnas.1618239114 28507138 PMC5465928

[B22] Hadjipanayi E. Mudera V. Brown R. A. (2009). Close dependence of fibroblast proliferation on collagen scaffold matrix stiffness. J. Tissue Eng. Regen. Med. 3, 77–84. 10.1002/term.136 19051218

[B23] Hartman C. D. Isenberg B. C. Chua S. G. Wong J. Y. (2016). Vascular smooth muscle cell durotaxis depends on extracellular matrix composition. Proc. Natl. Acad. Sci. USA. 113, 11190–11195. 10.1073/pnas.1611324113 27647912 PMC5056055

[B24] Hartman C. D. Isenberg B. C. Chua S. G. Wong J. Y. (2017). Extracellular matrix type modulates cell migration on mechanical gradients. Exp. Cell Res. 359, 361–366. 10.1016/j.yexcr.2017.08.018 28821395 PMC5603420

[B25] Herron T. J. Rocha A. M. Campbell K. F. Ponce-Balbuena D. Willis B. C. Guerrero-Serna G.. (2016). Extracellular matrix–mediated maturation of human pluripotent stem cell–derived cardiac monolayer structure and electrophysiological function. Circ. Arrhythm. Electrophysiol. 9, 1–12. 10.1161/CIRCEP.113.003638 27069088 PMC4833010

[B26] Hersch N. Wolters B. Dreissen G. Springer R. Kirchgeßner N. Merkel R.. (2013). The constant beat: cardiomyocytes adapt their forces by equal contraction upon environmental stiffening. Biol. Open 2, 351–361. 10.1242/bio.20133830 23519595 PMC3603417

[B27] Higuchi S. Lin Q. Wang J. Lim T. K. Joshi S. B. Anand G. S.. (2013). Heart extracellular matrix supports cardiomyocyte differentiation of mouse embryonic stem cells. J. Biosci. Bioeng. 115, 320–325. 10.1016/j.jbiosc.2012.10.004 23168383 PMC5330950

[B28] Hirt U. A. Waizenegger I. C. Schweifer N. Haslinger C. Gerlach D. Braunger J.. (2018). Efficacy of the highly selective focal adhesion kinase inhibitor BI 853520 in adenocarcinoma xenograft models is linked to a mesenchymal tumor phenotype. Oncogenesis 7:21. 10.1038/s41389-018-0032-z 29472531 PMC5833389

[B29] Holle A. W. Tang X. Vijayraghavan D. Vincent L. G. Fuhrmann A. Choi Y. S.. (2013). *In situ* mechanotransduction via vinculin regulates stem cell differentiation. Stem Cells 31, 2467–2477. 10.1002/stem.1490 23897765 PMC3833960

[B30] Ingber D. E. (2003). Mechanosensation through integrins: cells act locally but think globally. Proc. Natl. Acad. Sci. 100, 1472–1474. 10.1073/pnas.0530201100 12578965 PMC149854

[B31] Israeli-Rosenberg S. Manso A. M. Okada H. Ross R. S. (2014). Integrins and integrin-associated proteins in the cardiac myocyte. Circ. Res. 114, 572–586. 10.1161/CIRCRESAHA.114.301275 24481847 PMC3975046

[B32] Jacot J. G. McCulloch A. D. Omens J. H. (2008). Substrate stiffness affects the functional maturation of neonatal rat ventricular myocytes. Biophys. J. 95, 3479–3487. 10.1529/biophysj.107.124545 18586852 PMC2547444

[B33] Jahn D. Schramm S. Schnölzer M. Heilmann C. J. de Koster C. G. Schütz W.. (2012). A truncated lamin A in the Lmna -/- mouse line: implications for the understanding of laminopathies. Nucleus. 3, 463–474. 10.4161/nucl.21676 22895093 PMC3474667

[B34] Janmey P. A. Miller R. T. (2011). Mechanisms of mechanical signaling in development and disease. J. Cell Sci. 124, 9–18. 10.1242/jcs.071001 21172819 PMC3001405

[B35] Kaushik G. Spenlehauer A. Sessions A. O. Trujillo A. S. Fuhrmann A. Fu Z.. (2015). Vinculin network–mediated cytoskeletal remodeling regulates contractile function in the aging heart. Sci. Transl. Med. 7:292ra99. 10.1126/scitranslmed.aaa5843 26084806 PMC4507505

[B36] Kilian K. A. Bugarija B. Lahn B. T. Mrksich M. (2010). Geometric cues for directing the differentiation of mesenchymal stem cells. Proc. Natl. Acad. Sci. 107, 4872–4877. 10.1073/pnas.0903269107 20194780 PMC2841932

[B37] Kostetskii I. Li J. Xiong Y. Zhou R. Ferrari V. A. Patel V. V.. (2005). Induced deletion of the N-Cadherin gene in the heart leads to dissolution of the intercalated disc structure. Circ. Res. 96, 346–354. 10.1161/01.RES.0000156274.72390.2c 15662031

[B38] Lee B. Y. Timpson P. Horvath L. G. Daly R. J. (2015). Pharmacology and therapeutics FAK signaling in human cancer as a target for therapeutics. Pharmacol. Ther. 146, 132–149. 10.1016/j.pharmthera.2014.10.001 25316657

[B39] Lee H. P. Gu L. Mooney D. J. Levenston M. E. Chaudhuri O. (2017). Mechanical confinement regulates cartilage matrix formation by chondrocytes. Nat. Mater. 16, 1243–1251. 10.1038/nmat4993 28967913 PMC5701824

[B40] Lee H. P. Stowers R. Chaudhuri O. (2019). Volume expansion and TRPV4 activation regulate stem cell fate in three-dimensional microenvironments. Nat. Commun. 10:529. 10.1038/s41467-019-08465-x 30705265 PMC6355972

[B41] Li J. Minami I. Shiozaki M. Yu L. Yajima S. Miyagawa S.. (2017). Human pluripotent stem cell-derived cardiac tissue-like constructs for repairing the infarcted myocardium. Stem Cell Rep. 9, 1546–1559. 10.1016/j.stemcr.2017.09.007 29107590 PMC5829319

[B42] Li Y. Shi X. Tian L. Sun H. Wu Y. Li X. (2016). AuNP–collagen matrix with localized stiffness for cardiac-tissue engineering: enhancing the assembly of intercalated discs by ?1-integrin-mediated signaling. Adv. Mater. 28, 10230–10235. 10.1002/adma.201603027 27723133

[B43] Lo C. M. Wang H. B. Dembo M. Wang Y. L. (2000). Cell movement is guided by the rigidity of the substrate. Biophys. J. 79, 144–152. 10.1016/S0006-3495(00)76279-5 10866943 PMC1300921

[B44] Loessner D. Meinert C. Kaemmerer E. Martine L. C. Yue K. Levett P. A.. (2016). Functionalization, preparation and use of cell-laden gelatin methacryloyl-based hydrogels as modular tissue culture platforms. Nat. Protoc. 11, 727–746. 10.1038/nprot.2016.037 26985572

[B45] Lv P. C. Jiang A. Q. Zhang W. M. Zhu H. L. (2018). FAK inhibitors in cancer, a patent review. Expert Opin. Ther. Pat. 28, 139–145. 10.1080/13543776.2018.1414183 29210300

[B46] Lyon R. C. Zanella F. Omens J. H. Sheikh F. (2015). Mechanotransduction in cardiac hypertrophy and failure. Circ. Res. 116, 1462–1476. 10.1161/CIRCRESAHA.116.304937 25858069 PMC4394185

[B47] Major L. G. Choi Y. S. (2018). Developing a high-throughput platform to direct adipogenic and osteogenic differentiation in adipose-derived stem cells. J. Tissue Eng. Regen. Med. 12, 2021–2028. 10.1002/term.2736 30053766

[B48] Mariman E. C. Wang P. (2010). Adipocyte extracellular matrix composition, dynamics and role in obesity. Cell. Mol. Life Sci. 67, 1277–1292. 10.1007/s00018-010-0263-4 20107860 PMC2839497

[B49] McBeath R. Pirone D. M. Nelson C. M. Bhadriraju K. Chen C. S. (2004). Cell shape, cytoskeletal tension, and RhoA regulate stemm cell lineage commitment. Dev. Cell 6, 483–495. 10.1016/S1534-5807(04)00075-9 15068789

[B50] McCain M. L. Yuan H. Pasqualini F. S. Campbell P. H. Parker K. K. (2014). Matrix elasticity regulates the optimal cardiac myocyte shape for contractility. Am. J. Physiol. Heart Circ. Physiol. 306, H1525–H1539. 10.1152/ajpheart.00799.2013 24682394 PMC4042196

[B51] Mezzano V. Sheikh F. (2012). Cell-cell junction remodeling in the heart: possible role in cardiac conduction system function and arrhythmias? Life Sci. 90, 313–321. 10.1016/j.lfs.2011.12.009 22227473 PMC3488940

[B52] Nawata J. (1999). Differential expression of ?1, ?3 and ?5 integrin subunits in acute and chronic stages of myocardial infarction in rats. Cardiovasc. Res. 43, 371–381. 10.1016/S0008-6363(99)00117-0 10536667

[B53] Nielsen S. H. Mouton A. J. DeLeon-Pennell K. Y. Genovese F. Karsdal M. Lindsey M. L. (2017). Understanding cardiac extracellular matrix remodeling to develop biomarkers of myocardial infarction outcomes. Matrix Biol. 75–76, 1–15. 10.1016/j.matbio.2017.12.001 29247693 PMC6002886

[B54] Pandey P. Hawkes W. Hu J. Megone W. V. Gautrot J. Anilkumar N.. (2018). Cardiomyocytes sense matrix rigidity through a combination of muscle and non-muscle myosin contractions. Dev. Cell 44, 326–336.e3. 10.1016/j.devcel.2017.12.024 29396114 PMC5807060

[B55] Rape A. D. Guo W. H. Wang Y. L. (2011). The regulation of traction force in relation to cell shape and focal adhesions. Biomaterials 32, 2043–2051. 10.1016/j.biomaterials.2010.11.044 21163521 PMC3029020

[B56] Ribeiro M. C. Tertoolen L. G. Guadix J. A. Bellin M. Kosmidis G. D'Aniello C.. (2015). Functional maturation of human pluripotent stem cell derived cardiomyocytes *in vitro*- correlation between contraction force andelectrophysiology. Biomaterials 51, 138–150. 10.1016/j.biomaterials.2015.01.067 25771005

[B57] Rosales A. M. Vega S. L. DelRio F. W. Burdick J. A. Anseth K. S. (2017). Hydrogels with reversible mechanics to probe dynamic cell microenvironments. Angew. Chem. Int. Ed. 56, 12132–12136. 10.1002/anie.201705684 28799225 PMC5668133

[B58] Ruedinger F. Lavrentieva A. Blume C. Pepelanova I. Scheper T. (2014). Hydrogels for 3D mammalian cell culture: a starting guide for laboratory practice. Appl. Microbiol. Biotechnol. 99, 623–636. 10.1007/s00253-014-6253-y 25432676

[B59] Sessions A. O. Min P. Cordes T. Weickert B. J. Divakaruni A. S. Murphy A. N.. (2018). Preserved cardiac function by vinculin enhances glucose Oxidation and extends health- and life-span. APL Bioeng. 2:036101. 10.1063/1.5019592 30105314 PMC6086353

[B60] Sheikh F. Ross R. S. Chen J. (2009). Cell-cell connection to cardiac disease. Trends Cardiovasc. Med. 19, 182–190. 10.1016/j.tcm.2009.12.001 20211433 PMC3601820

[B61] Sullivan T. Escalante-Alcalde D. Bhatt H. Anver M. Bhat N. Nagashima K.. (1999). Loss of A-type lamin expression compromises nuclear envelope integrity leading to muscular dystrophy. J. Cell Biol. 147, 913–919. 10.1083/jcb.147.5.913 10579712 PMC2169344

[B62] Swaminathan V. Waterman C. M. (2016). The molecular clutch model for mechanotransduction evolves. Nat. Cell Biol. 18, 459–461. 10.1038/ncb3350 27117328 PMC6792288

[B63] Swift J. Discher D. E. (2014). The nuclear lamina is mechano-responsive to ECM elasticity in mature tissue. J. Cell Sci. 127, 3005–3015. 10.1242/jcs.149203 24963133 PMC4095853

[B64] Swift J. Ivanovska I. L. Buxboim A. Harada T. Dingal P. C. Pinter J.. (2013). Nuclear lamin-A scales with tissue stiffness and enhances matrix-directed differentiation. Science 341:1240104. 10.1126/science.1240104 23990565 PMC3976548

[B65] Thiele J. Ma Y. Bruekers S. M. Ma S. Huck W. T. (2014). 25th anniversary article: designer hydrogels for cell cultures: a materials selection guide. Adv. Mater. 26, 125–148. 10.1002/adma.201302958 24227691

[B66] Urban M. W. Pislaru C. Nenadic I. Z. Kinnick R. R. Greenleaf J. F. (2013). Measurement of viscoelastic properties of *in vivo* swine myocardium using lamb wave dispersion ultrasound vibrometry (LDUV). IEEE Trans. Med. Imaging 32, 247–261. 10.1109/TMI.2012.2222656 23060325 PMC3562367

[B67] Vincent L. G. Choi Y. S. Alonso-Latorre B. del Álamo J. C. Engler A. J. (2013). Mesenchymal stem cell durotaxis depends on substrate stiffness gradient strength. Biotechnol. J. 8, 472–484. 10.1002/biot.201200205 23390141 PMC3749305

[B68] Wang H. B. Dembo M. Wang Y. L. (2000). Substrate flexibility regulates growth and apoptosis of normal but not transformed cells. Am. J. Physiol. Cell Physiol. 279, C1345–C1350. 10.1152/ajpcell.2000.279.5.C1345 11029281

[B69] Wang Z. Golob M. J. Chesler N. C. (2016). Viscoelastic Properties of Cardiovascular Tissues. In Viscoelastic and Viscoplastic Materials, 2:64 InTech. 10.5772/64169

[B70] Wineman A. S. Rajagopal K. R. (2000). Mechanical Response of Polymers: An Introduction. Cambridge: Cambridge University Press.

[B71] Yahalom-Ronen Y. Rajchman D. Sarig R. Geiger B. Tzahor E. (2015). Reduced matrix rigidity promotes neonatal cardiomyocyte dedifferentiation, proliferation and clonal expansion. Elife 4, 1–18. 10.7554/eLife.07455 26267307 PMC4558647

[B72] Ye N. Verma D. Meng F. Davidson M. W. Suffoletto K. Hua S. Z. (2014). Direct observation of α-actinin tension and recruitment at focal adhesions during contact growth. Exp. Cell Res. 327, 57–67. 10.1016/j.yexcr.2014.07.026 25088253 PMC4153383

[B73] Young J. L. Engler A. J. (2011). Hydrogles with time-dependent material properties enhance cardiomyocyte differentiation *in vitro*. Biomaterials 32, 1002–1009. 10.1016/j.biomaterials.2010.10.020 21071078 PMC3000555

[B74] Yu O. M. Miyamoto S. Brown J. H. (2016). Myocardin-related transcription factor A and yes-associated protein exert dual control in G protein-coupled receptor- and RhoA- mediated transcriptional regulation and cell proliferation. Mol. Cell. Biol. 36, 39–49. 10.1128/MCB.00772-15 26459764 PMC4702594

